# On the nature of voters’ coalition preferences

**DOI:** 10.1080/17457289.2016.1270286

**Published:** 2016-12-26

**Authors:** Carolina Plescia, Julian Aichholzer

**Affiliations:** ^a^ Department of Government, University of Vienna, Vienna, Austria

## Abstract

An expanding literature indicates that in multiparty systems with coalition governments, citizens consider the post-electoral bargaining process among parties when casting their vote. Yet, we know surprisingly little about the *nature* of voters’ coalition preferences. This paper uses data from the Austrian National Election Study to examine the determinants as well as the independence of preferences for coalitions as political object. We find that coalition preferences are strongly informed by spatial considerations; but additional non-ideological factors, such as party and leader preferences, also play a fundamental role. We also find that coalitions enjoy a certain degree of independence from other objects of vote choice and they do not always represent a simple average score on the feeling thermometer of the constituent parties. There are, however, substantial differences among voters, with party identifiers and those with extreme ideology being less likely to consider coalitions as separate entities from their component parties.

## 1. Introduction

In countries where no single party has a majority in the legislature, discussion and speculations about potential coalition governments are standard elements of the electoral campaign (Bowler, Karp, and Donovan 2010; Hobolt and Karp 2010). Coalition discourse characterizes the political debate not only where coalition governments are common, but, more recently, also in countries like the United Kingdom, where coalition governments are a new experience for voters. In recent years, electoral research has started to investigate whether voters consider potential coalitions that could form after the election when casting their vote. The existence of ‘coalition-targeted voting’ seems well-established now: where coalition governments are the norm, voters consider the coalition formation process and coalition bargaining when casting their vote (Blais et al. 2006; Duch, May, and Armstrong 2010; Gschwend 2007; Indridason 2011; Kedar 2005).

The presence of coalitions as an integral part of the decision-making calculus of voters should have consequences for the way people vote. If coalition-oriented, voters may ‘abandon’ their *most preferred party* and lend support to another party to achieve a specific coalition outcome: research has found that strategic voting is directed at coalitions rather than at parties (Bargsted and Kedar 2009; Meffert and Gschwend 2010). For these voters, at least, coalition preferences seem to have an independent effect on vote choice, ‘over and above their views about parties, the leaders and their ideological orientations’ (Blais et al. 2006, 702).

Despite the increasing prominence that coalition-oriented voting is gaining in the literature, we know surprisingly little about the *nature of voters’ coalition preferences*. In fact, as of today, only very few studies have examined why voters prefer some combination of parties as coalition governments over others. Recent findings suggest that coalitions are salient evaluation objects of vote choice which appear to enjoy a certain degree of independence from party and leader preferences (Debus and Müller 2014; Falcó-Gimeno 2012; Meffert and Gschwend 2012).

This paper builds on this recent research to study the determinants of voters’ coalition preferences as well as the independence of these preferences from party preferences. In the following we ask two main research questions: (1) What determines coalition preferences? (2) Are coalitions just a sum of their parties or can voters have preferences for coalitions that differ from the preferences they have for the coalition’s component parties?

This research employs data from the 2013 Austrian National Election Study (AUTNES). These data allow us to parse several potential roots of coalition preferences beyond forecasted policy outcomes. Our study represents one of the first attempts to unpack coalition preferences into individual components. Two main results emerge from this analysis. First, while ideological proximity exerts a rather strong impact it is not the sole determinant of coalition preferences. Other considerations also inform coalition attitudes such as party and leader preferences. Second, it seems that coalition preferences do not always represent a simple average score on the feeling thermometer of the constituent parties. Rather coalitions appear to be discrete political objects that voters relate to. In this regard, the results indicate variation on coalition evaluations across individuals with party identifiers and those with extreme ideological positions being less likely to consider coalitions as separate entities from their component parties.

We extend previous knowledge by showing that coalition preferences matter beyond preferences for their component parties. This has consequences for party politics more generally. In fact, if coalitions are important per se, parties may face particularly heavy electoral sanctions if they do not take coalition preferences into account when openly considering coalition options before elections and when making decisions about the partisan composition of the next government. Below we begin by reviewing the literature, outlining the questions to be answered and the hypotheses to be tested. Then, we describe the context and the data used to conduct empirical tests. Finally, we delineate the results and discuss their implications.

## 2. What determines coalition preferences?

Until now, most of the literature on voting behavior has focused on two political objects: parties and candidates; voting research has not dealt much with coalition evaluations and their impact on vote choice. Recently, however, a growing body of literature has devoted attention to coalitions as an integral part of the decision-making calculus of voters (Bowler et al. 2010; Duch et al. 2010; Kedar 2005). Several studies demonstrate the predictive value of coalition preferences over and above that of parties, as voters’ expectations of what will happen after the elections influence how citizens vote (Bargsted and Kedar 2009; Blais et al. 2006; Meffert and Gschwend 2010). Despite a growing literature on the subject, the very basic question about the nature and independence of voters’ coalition preferences has received little attention. However, if voters cast coalition-targeted ballots, it becomes crucial to understand voters’ coalition attitudes.

### 2.1. Policy considerations

A fundamental component of coalition preferences is based on the seminal work by Downs (1957). In the Downsian framework, voters, parties, and candidates are assumed to hold positions in the ideological space and the utility of voters is determined by the distance between the voter and the political object, that being the party or the candidate (Hinich and Munger 1994). To capture the impact of the post-election coalition bargaining process on vote choice, Duch et al. (2010, 716) alter the proximity voting calculation to include not only the distance between the party platform and the voter, but also the distance between the voter and the potential government policy that will be enacted with that party in government (see also Kedar 2005). The authors find that party support is conditional on the overall coalition bargaining outcomes that occur when the election is over. Hence, it is the distance between the voter and the overall position of the potential coalition that should matter. Falcó-Gimeno (2012) finds, for instance, that Spanish voters’ coalition preferences are largely determined by ideological proximity, especially on the left–right axis.

Debus and Müller (2014), however, argue that not only the expected policy position of a coalition government is decisive for coalition preferences, but also the distances to the single coalition partners. This idea is akin to models of coalition politics and cabinet governance, such as veto player theory (Tsebelis 2002) and the ministerial discretion model (Laver and Shepsle 1990, 1996), which argue that the utility associated with a potential government is a function of the voter’s ideal position and the policy positions of *each* of the parties involved. Accordingly, voter *i* is likely to prefer coalitions that only include parties that are close to *i*’s policy position.

Not only the distance voter–party and voter–coalition should matter, but also the distance between the two coalition partners themselves, that is, programmatic heterogeneity. Golder (2006) shows that the successful formation of a pre-electoral coalition is largely dependent upon the ideological distance between the coalition partners, whereas ideological (programmatic) congruence should mitigate frictions among coalition members and enhance coalition effectiveness and competence (see also Glasgow, Golder, and Golder 2012; Martin and Stevenson 2001). A similar logic can be electorally relevant, as well. Voters are less likely to prefer coalitions if they expect many policy concessions to be made (Gschwend and Hooghe 2008). Indeed, if voters are mindful of coalition effectiveness and competence, the more congruent the ideological position of the coalition partners, the more likely voters will prefer them as future government (Plescia 2016). This expectation is echoed in Debus and Müller (2014), who find that voters favor coalition governments with a low degree of internal programmatic heterogeneity.

### 2.2. Non-policy considerations

While policy preferences are a key component of coalition preferences, there are additional considerations at play. In fact, electoral preferences are rarely determined by policy alone (e.g. Green and Hobolt 2008). Theoretically, it has been postulated that coalition preference follows, first of all, from *party preference* (Pappi and Thurner 2002). Coalitions are constituted by parties, and voters vote for parties not coalitions, at least not directly, thus feelings for parties should obviously matter to explain coalition preferences (Meffert and Gschwend 2012). Simply put, voters should like coalitions that are composed of parties they like so that a *similarity* score of the preferences for those parties should have a positive effect on the overall coalition preference.

Yet, a simple similarity score between the parties’ preferences is valid only under the assumption that positive and negative feelings exert equally strong impact on political judgments. While this is an empirical question we address below, *a priori* there are good reasons to think that the effects of negative and positive evaluations may be asymmetrical in magnitude (Cacioppo, Gardner, and Berntson 1997; Holbrook et al. 2001; Lavine, Johnston, and Steenbergen 2012, 64). Indeed, individuals tend to think more about and place greater credibility on negative assessments than on positive ones (see Lau 1982; Soroka 2014; Wagner and Meyer 2015) leading to negative assessments having more influence on overall evaluations than positive assessments (Soroka 2014). Hence, while overall, we expect that voters will like a coalition more when it includes the most liked party and reject those coalitions including the parties they dislike, we also expect that the inclusion of a disliked party will have a greater effect than the inclusion of the most liked party.

Given an increased attention to the political protagonists instead of political issues in contemporary politics (Caprara and Zimbardo 2004), personal characteristics of the *party leaders* might have an influence on coalition preferences as well. It seems straightforward that *similarity* of preferences for the leading candidates also influences coalition preferences, especially in contexts like Austria, Germany, and Italy where the leaders of the main parties enjoy considerable visibility during the elections campaigns. Still, attitudes towards the leader of the larger coalition partner (and future prime minister) should bear disproportional influence on coalition preferences when compared to the preferences for the leader of the junior coalition partner as leaders of the main parties enjoy more visibility during the elections (Bowler, Gschwend, and Indridason 2014).

## 3. Are coalitions just a sum of their parties?

The explanatory factors related to ideology and affect we just discussed require the assumption that voters possess preferences for coalitions, parties and leaders that are to a large extent compatible with each other. From a psychological perspective, both parties and coalitions can be conceptualized as evaluative objects that can either be related to each other or remain independent (Meffert and Gschwend 2012). Yet, while parties and leaders are real and salient entities, coalitions are hypothetical constructs, exception made for currently existing coalitions or coalitions that have been formed in the past. In many multiparty settings, voters have experienced coalition politics for decades and in most of these countries single parties have never had a majority in the legislature. This means that voters have had the time to learn, cope and possibly form real attitudes towards those coalition arrangements that represent historical regularities and common patterns (Armstrong and Duch 2010; Banaszak and Doerschler 2012; Fortunato and Stevenson 2013). Hence, there is a possibility that, over time, coalitions may have developed into a meaningful political object for some voters who have hence come to develop specific coalition preferences over those of parties (Huber 2014).

Recent findings may be interpreted as providing support for this claim. Specifically, a considerable body of work on coalition politics assumes that the policy position of coalition governments represents a weighted average of party positions, with the weights given by party size (e.g. Gamson 1961; Martin and Vanberg 2014). However, Bowler et al.’s (2014) study indicates that voters differ substantially in their perceptions of coalition policy platforms and, more importantly, their perceptions differ from the average of the perceived party policy positions. While this finding may be interpreted by resorting to the argument that voters do not understand the system in which they live; Meyer and Strobl (2016) find no consistent effect of political knowledge on voters’ uncertainty when evaluating coalition policy positions, which should in fact refute this pessimistic argument.

If coalition evaluations were only derivations from party evaluations, the rating of a specific coalition should be a convex combination of the ratings of the parties that are part of this coalition. That is, the rating of the preferred party should always be higher than the ratings of the preferred coalition that consists of this party and another party. If, however, coalitions are discrete political objects that voters relate to, voters may also rate a coalition higher or lower than the parties it is composed of (Huber 2014).

In this regard, the incumbent coalition in particular should be more likely to be seen as a meaningful political object and display scores of coalition preferences that should not just reflect those of the component parties, if for no other reason than because voters have experience of that coalition in the recent past. In addition, just like individual candidates benefit from factors like name recognition, parties and coalitions benefit from exposure. The most frequently studied indicator is the incumbency status (e.g. Carey, Niemi, and Powell 2000; Clark 2009) which is a shorthand indicator for a number of factors like name recognition due to media exposure. With a specific view on coalitions, this is reflected in the empirical regularity uncovered by Debus and Müller (2014) who show that coalitions which were frequently put into place in previous electoral cycles generate familiarity among voters that translates into a preference surplus.

The existing literature also provides ground to expect differences among voters. We posit here attention to three key individual-level factors that may indeed have an influence on coalition evaluations: party identification, voters’ ideology and political sophistication. First and foremost, in the case of voters who identify with a party or who strongly prefer a party above all the others, parties should function as the principal voting cue (Campbell and Miller 1957). Party identification can be thought of as a heuristic voters use to make sense of the complexity of the political world (Lau and Redlawsk 2001). Partisanship affects how individuals evaluate parties and thus acts as a ‘perceptual screen’ in how they react to new information and form opinions (Taber and Lodge 2006; Zaller 1992). Consequently, we would expect that the more party-centered the voter, the less likely he or she will be to think in terms of coalitions rather than parties.

Moving to voters’ ideology, we test whether ideological stances have an effect on coalition considerations. Decades of divided government in the US led many scholars to address the question why some voters split their ballot by selecting a Republican for one office and a Democrat for another (e.g. Alvarez and Schousen 1993). The ‘policy-balancing hypothesis’ (Fiorina 1996) links to the idea that moderate voters are more likely to prefer divided government as to balance the power of the two parties in representative structures (e.g. Alesina and Rosenthal 1995). If centrist voters are more likely than ideologues to cast a split ballot, one would have evidence consistent with policy-balancing theories (Burden and Kimball 2004, 100). Applying this argument to multiparty contexts is more complex however. The general idea is that coalition considerations can be sensitive to respondents’ ideology and in this regard extreme voters may be less sensitive to coalitions as policy compromise than their moderate counterparts (Bargsted and Kedar 2009; Fortunato and Stevenson 2013). Hence, we test whether or not ideological extremism has a negative effect on the probability of ranking that coalition independently from the component parties.

Last but not least, reasoning about coalitions is thought to be more complex than reasoning about parties, because voters vote for parties not coalitions, at least not directly. Given that parties do not campaign together on a common election platform, all voters need to integrate information on the individual parties to form expectations on coalitions and usually it is hypothesized that well-informed voters would find it easier to perform these tasks (Meyer and Strobl 2016). Thus, at the individual level, if coalition evaluations are the realm of the sophisticated and more educated, we would expect these respondents to be more likely to rate a coalition independently from the parties it is comprised of than their respective counterparts. Our hypotheses on the determinants of coalition preferences are summarized in Table 1.

**Table 1.** Hypotheses.

Effect on:			
Coalition preferences		Independence of coalition preferences	
Hypotheses	Expected sign	Hypotheses	Expected sign
Distance voter–coalition	Negative	Incumbent coalition	Positive
Distance voter–largest party	Negative	Party identification	Negative
Distance voter–smallest party	Negative	Education	Positive
Programmatic heterogeneity of parties	Negative	Political knowledge	Positive
Parties preference similarity	Positive	Extreme ideology	Negative
Inclusion of top party	Positive		
Inclusion of bottom party	Negative		
Leaders preference similarity	Positive		
Preference leader of largest party	Positive		

## 4. Data and variables

We test our propositions using data from Austria. First, Austria has a long history of coalition governments (Müller 2005). Elections simply set the stage for post-election negotiations that determine the composition of the government, most often between the two mainstream parties, the Austrian People’s Party (ÖVP) and the Social Democratic Party of Austria (SPÖ). Beside these two dominant parties, Austria’s party system is characterized by another main player, the far-right Freedom Party of Austria (FPÖ) and, in some federal provinces, the Greens, further complicating government formation. In addition, existing works found evidence of coalition-targeting voting in Austria (Meffert and Gschwend 2010; Meffert et al. 2011).

Second, the 2013 AUTNES (Kritzinger et al. 2014a) provides high-quality pre- and post-electoral data, not usually available in other countries, which allows us to delve into micro-level relationships of interest. The AUTNES Pre- and Post-Panel Study (Kritzinger et al. 2014b) was carried out in two waves: in total 3266 respondents completed the face-to-face interviews conducted before the elections (AAPOR response rate: 61.8%). Unusual for an election survey, respondents were asked to rate themselves, the parties and coalitions both in terms of preferences and ideological stances. The AUTNES questionnaire included questions on four possible coalition outcomes: a so-called grand coalition between the two mainstream parties (SPÖ–ÖVP), two coalitions formed by the SPÖ and either the Greens (SPÖ–Greens) or the far-right party, the FPÖ (SPÖ–FPÖ), respectively, and finally a coalition formed by the ÖVP and the FPÖ (ÖVP–FPÖ) (The Online Appendix shows descriptive statistics for all variables used in the analysis).

Starting with ideological positions for voters, parties and coalitions we use a general 0 (extreme left) to 10 (extreme right) left–right scale.^1^ We then use these positional questions to calculate the distances voter–party, party–party, and voter–coalition. Thus, in order to measure the perceived distance between the voter and the coalition, we employ the left–right scale asked for coalitions directly.^2^

We measure preferences for both parties and leaders using the thermometer scale from 0 (‘do not prefer it at all’) to 10 (‘very much prefer it’). Preferences similarity is measured straightforwardly taking the inverse of the absolute difference between the preferences for the two coalition partners for the party similarity variable and between the two party leaders for the leader similarity variable.^3^ In other words, larger values of the variable indicate more similar preferences. We further establish, for each respondent, a rank of parties using again the thermometer preference question. Using this rank of party, we build two dummy variables: the first variable takes a value of 1 when the coalition includes the top-ranked party by that specific individual and 0 otherwise; a second dummy variable takes a value of 1 when the coalition includes the bottom-ranked party and 0 otherwise. Note that, when voters like (or dislike) more than one party in equal measure, the dummy variables take a value of 0 when the coalition includes either one of these parties. On the other hand, *partisanship* indicates whether the respective party is the party a respondent ‘feels closest to’ employing the question: ‘Generally speaking, do you feel close to a particular party?’. We recode this variable to take a value of 1 if the respondent feels close to that party and 0 otherwise.

Education ranges from 0 (low level education) to 1 (highest level of education) with an intermediate value of 0.5. Political knowledge represents an additive scale measured by a battery of seven questions for general political knowledge. Ideological extremism is measured in the following way: for every respondent, we take the absolute distance between her placement and the center of the left/right scale. Hence higher values indicate more extreme positions. As explained in the next section, in our models, we include a linear transformation of the variables: education, political knowledge, and ideological extremism.

## 5. Empirical results: the determinants of coalition preferences

In this first step, our dependent variable is the strength of coalition preference measured on a scale from 0 (‘do not prefer it at all’) to 10 (‘very much prefer it’) for four different coalitions: SPÖ–Greens, SPÖ–ÖVP, ÖVP–FPÖ, and SPÖ–FPÖ. As one can assume that parties’ and coalitions’ evaluations have effects on all potential coalitions and not only on particular ones, modeling all potential coalitions in one model offers a general picture of the effect of these evaluations. Thus, the data set is expanded by the number of coalitions available in our survey. In this ‘stacked’ dataset, coalition preferences serve as dependent variable, with each respondent contributing four observations, one for each of the coalitions available in our survey. Therefore, after stacking, the unit of analysis are not respondents, but responses (individual × coalitions). The structure of this transformed data is best described as hierarchical, with responses (level 2) nested within respondents making these assessments (level 1). Because of this, and due to the fact that the stacking procedure artificially increases the number of observations and may cause concerns over the independence of errors (Pardos-Prado and Dinas 2010), we run a hierarchical multiple linear regression with random intercepts specified at the respondent level. We also include fixed effects to account for correlated error across coalitions.^4^

This reshaping changes the unit of analysis from the number of individual respondents to responses (Van der Eijk et al. 2006). Because of this, an independent variable in order to be included in the analysis needs to be also defined in terms of bivariate relationships between the chooser (i.e. respondent) and the object of evaluation (i.e. coalition). Some independent variables such as the distance voter–coalition are already defined as respondent × coalition-specific relationships. Other variables instead, like education, political knowledge, and ideological extremism, need to be re-conceptualised as proximity measure in order to capture the empirical relationship between voters and coalitions. To this end, we apply the so-called *y-hat* approach (van der Eijk and Franklin 1996). The specific procedure for doing so is based on multivariate regressions (run separately for each coalition) on the specific predictors: predicted values (*y-hats*) are then centered on their means and saved as scores for use in the later analysis as coalition–respondent-specific predictors. The *b* coefficient for a specific predictor, for example, political knowledge, only expresses the importance of that variable in general.^5^

Table 2 presents all models. We first run models including either only policy or non-policy factors, we then run complete models. The baseline models (M1 and M2) include only policy evaluations. M1 includes voters’ distance to the overall coalition position; M2 includes voters’ distance to the two coalition partners separately, which allows us to discover which one of the two coalition partners has the strongest effect on the overall coalition score. The results indicate that coalition preferences are strongly influenced by ideological evaluations with negative effects throughout: these negative coefficients mean that the more distant citizens are to a political object, the less they prefer that object. We find that not only the distance to the overall coalition is important but also the separate distances to the two coalition partners with the distance to the smaller party having an even larger impact in explaining coalition preferences. While this finding is quite surprising on its face, it echoes the findings from a related strand of literature: concerning voters’ perception of coalition positions, Bowler et al. (2014) find that voters perceive smaller coalition parties having disproportional influence on coalition policy.^6^ Programmatic heterogeneity, that is the distance party–party within the coalition, has a significant effect on coalition evaluations but the direction of this effect is not consistent across M1 and M2.

**Table 2:** . The determinants of coalition preferences: hierarchical linear regression models.

	Dependent variable: coalition preference					
	(M1)	(M2)	(M3)	(M4)	(M5)	(M6)
Distance to coalition	−0.57*** (0.01)			−0.36*** (0.01)		−0.24*** (0.02)
Programmatic heterogeneity	0.05*** (0.02)	0.12*** (0.02)		−0.03* (0.01)	0.08*** (0.01)	0.05*** (0.01)
Distance largest party		−0.22*** (0.02)			−0.05** (0.02)	0.04* (0.02)
Distance smaller party		−0.52*** (0.01)			−0.34*** (0.01)	−0.24*** (0.02)
Parties preference similarity			0.14*** (0.02)	0.12*** (0.01)	0.13*** (0.01)	0.12*** (0.01)
Coalition includes top-ranked party			2.27*** (0.06)	2.02*** (0.06)	1.88*** (0.06)	1.86*** (0.06)
Coalition includes bottom-ranked party			−1.33*** (0.07)	−1.15*** (0.06)	−0.96*** (0.06)	−0.96*** (0.06)
Leaders preference similarity			0.06*** (0.01)	0.05*** (0.01)	0.06*** (0.01)	0.06*** (0.01)
Preference leader of large party			0.26*** (0.01)	0.21*** (0.01)	0.25*** (0.01)	0.24*** (0.01)
Education (*y-hat*)	0.76*** (0.15)	0.48*** (0.14)	0.44*** (0.13)	0.33** (0.13)	0.21 (0.13)	0.23 (0.12)
Political knowledge (*y-hat*)	0.91 (0.50)	1.06* (0.48)	0.26 (0.45)	0.37 (0.43)	0.51 (0.43)	0.47 (0.42)
Extreme ideology (*y-hat*)	0.10 (0.08)	0.17* (0.07)	0.55*** (0.07)	0.08 (0.07)	0.15* (0.07)	0.02 (0.07)
*Reference (SPÖ–Green coalition):*						
SPÖ–ÖVP	0.26*** (0.07)	0.12 (0.07)	0.44*** (0.07)	0.23*** (0.06)	0.17** (0.06)	0.11 (0.06)
ÖVP–FPÖ	−1.10*** (0.07)	−0.94*** (0.07)	−0.54*** (0.07)	−0.46*** (0.07)	−0.40*** (0.07)	−0.40*** (0.06)
SPÖ–FPÖ	−1.71*** (0.09)	−1.62*** (0.08)	−1.06*** (0.07)	−1.11*** (0.08)	−1.06*** (0.08)	−1.12*** (0.07)
Intercept	5.67*** (0.07)	5.88*** (0.07)	0.73*** (0.15)	2.12*** (0.16)	1.82*** (0.16)	2.08*** (0.16)
*Random effects*						
Intercept variance, respondents	0.58	0.75	0.70	0.86	0.78	0.85
*N_*stacked (respondents × coalitions)	9689	9689	9689	9689	9689	9689
*N* (respondents)	2706	2706	2706	2706	2706	2706
Log likelihood	−23,301.8	−23,111.4	−22,397.2	−22,044.9	−22,009.7	−21,917.0
AIC	46,625.6	46,246.8	44,822.4	44,121.8	44,053.4	43,869.9
BIC	46,704.6	46,332.9	44,922.9	44,236.6	44,175.4	43,999.2

Notes: Standard errors in parentheses. *y-hat* variables are predicted values.

* *p *< .05, ** *p* < .01, *** *p* < .001.

M3 tests the effect of preference similarities for the two coalition partners, both in terms of parties and leaders, which we find to have a positive and statistically significant effect; however, preferences for the leader of the largest party and future chancellor exert the strongest positive effect. M3 also indicates that the inclusion of the most preferred party in the coalition has a strong and positive impact on coalition preferences; conversely the inclusion of the bottom-ranked party has a statistically significant negative impact. While both positive and negative attitudes are relevant, their effect is diverse and, contrary to expectations, negative evaluations appear to have a lower impact on coalition preferences than positive evaluations. Finally, M4, M5, and M6 bring all variables together; despite the effect of all independent variables decreasing somewhat, all the variables remain statistically significant.^7^ However, there are some interesting differences that are worth noting. First, the complete models tell us that the strongest effect is observed for the inclusion of the top- and bottom-ranked party; in addition, the affective component has the strongest effect especially with regard to the junior coalition partner. It is also worth mentioning that in some of the complete models, that is, M2, M5, and M6, the effect of programmatic heterogeneity remains significant but is it now positive instead of negative.^8^

As far as the *y-hat* variables are concerned, we find a significant effect of ideology extremism and education on coalition preferences, albeit not across all models. Overall, these results indicate that while ideological distances and preferences for parties and preferences for leaders are important to explain coalition preferences, unique coalition indicators such as coalition ideological position and coalition heterogeneity have an independent and unique contribution to explaining coalition preferences. That is, voters take coalition-specific factors into account when assessing coalitions apart from only evaluating the single parties involved.^9^

## 6. Empirical results: are coalitions just a sum of their parties?

In this final section, we examine the independence of preferences for coalitions as political object. We explained before that if coalition evaluations were only derivations from party evaluations, the rating of a specific coalition should be a convex combination of the ratings of the parties that are part of this coalition. If, however, coalitions are discrete political objects that voters relate to, voters may also rate a coalition higher or lower than the parties it is composed of. To examine this, we determine the top-scored party and the bottom-scored party for each specific coalition arrangement for each respondent and distinguish between respondents who score the coalition as high (low) as the top (bottom) party included in the coalition, those who score the top coalition somewhat in the middle, and those whose coalition preferences are below or above the score of the constituent parties.

Complete patterns are shown in Table 3.^10^ The incumbent grand coalition formed by the two largest parties, the SPÖ and the ÖVP, is the one most often scored above the top-ranked party and it is also the coalition receiving the highest evaluation across all respondents (mean = 4.7, SD = 2.9). It is worth noting that this is the only ‘real’ coalition among the ones examined here, that is, the coalition that was in power before the elections and the one with the highest associated likelihood to take place again after the elections. In turn, we find that the coalition formed by the SPÖ and the far-right FPÖ coalition is the one most often scored below the bottom-ranked party; this is the coalition receiving the lowest preference score across all respondents (mean = 2.2, SD = 2.6). Furthermore, it was also the coalition least likely to take place after the elections. Overall, we see that in only about half of the instances, the rating of a specific coalition is somewhat of a mean of the ratings of the constituent parties. Quite often coalitions score above or below the parties they are composed of.

**Table 3.** Patterns of coalition preferences (cell entries show column %).

	Coalition			
	SPÖ–Greens	SPÖ–ÖVP	ÖVP–FPÖ	SPÖ–FPÖ
Coalition score somewhat in the middle or as high (low) as top (bottom) party	57.8	60.2	66.1	61.7
Coalition score above top party	13.3	18.2	8.9	6.3
Coalition score below bottom party	28.9	21.7	24.9	32.0
*N* (*respondents*)	3010	3049	3006	2941

Who are the respondents that score the top-ranked coalition above or below the top-ranked party? Given that in this second instance, the dependent variable is trichotomous, reflecting the categories in Table 3, we run multinomial hierarchical logistic regression with individual-level random effects that take into account our stacked data matrix.^11^

In the previous discussion, we stressed that we expect individual-level differences by party identification, ideology, education, and political knowledge as well as differences across coalitions. Also, our model includes a variable measuring preference similarity for the coalition partners to control for the possibility of a higher likelihood of scoring a coalition independently from the component parties, the shorter the preference distance between the parties.

Starting from differences across coalitions, we hypothesized that the incumbent coalition would generate familiarity among voters translating into a preference surplus for that coalition. We find that the incumbency coalition scores more often above the top party, and less often below the bottom party. When it comes to party identification, we expect that identification with one of the coalition partners will have a negative effect on scoring the coalition above (below) the top (bottom) ranked party. In line with this expectation, Table 4 reflects a significant negative effect of party identification on the probability (i.e. making it less likely) of scoring the coalition below the bottom-ranked party but a positive effect of scoring the coalition above the top party.^12^

**Table 4.** Coalitions *vis*-à-*vis* parties: Hierarchical multinomial logit models.

Dependent variable: coalition score	Reference category: coalition as (weighted) average of the constituent parties	
	Above top party	Below bottom party
Party identification	0.62*** (0.08)	−0.21** (0.07)
Education (*y-hat*)	6.84*** (2.07)	4.38* (1.86)
Political knowledge (*y-hat*)	7.92** (2.47)	2.61 (1.65)
Extreme ideology (*y-hat*)	2.30* (0.93)	3.86*** (0.65)
Incumbent coalition	0.45*** (0.08)	−0.32*** (0.07)
Parties preference similarity	0.59*** (0.02)	0.38*** (0.02)
Intercept	−6.60*** (0.21)	−3.63*** (0.13)
*Random effects*		
Intercept variance, respondents	0.99	
*N*_stacked (respondents × coalitions)	9689	
*N* (respondents)	2706	
LL	−7562.6	
AIC	15,155.2	
BIC	15,262.9	

Note: Standard errors in parentheses. The reference category is having scored the coalition somewhat in the middle or as high (low) as top (bottom) party. *y-hat* variables are predicted values.

* *p* < .05, ** *p* < .01, *** *p* < .001.

With regard to ideology, education, and political knowledge, as previously discussed, the *b* coefficient for *y-hat* variables only expresses the importance of such a predictor in general. From Table 4, we see that overall the three variables have a significant effect on our dependent variable. To be able to interpret both the significance and the direction of these variables, Table S7 of the Online Appendix reports the effects of the same predictors in different stacks, that is, coalitions. There we see that, in line with the hypotheses, when significant, education increases the likelihood of scoring the coalition above the top-ranked, but also below the bottom-ranked party, that is, evaluating the coalition independently from parties from which it is comprised of. However, the impact of political knowledge seems to be reversed. With regard to extreme ideology, the results indicate that respondents with more extreme ideology tend to score a coalition above the top party or below the bottom party less often. This result suggests that people who are in the middle of the left/right ideological scale tend to be more likely to evaluate coalitions independently from parties from which it is comprised of than those positioning themselves at the extreme. Finally, when it comes to the effect of a variable measuring parties preference similarity, Table 4 shows that the variable has a positive impact on our dependent variable.

Two main results stand out from this analysis. First, while for a majority of voters coalition preferences represent somewhat of an average score of the feeling thermometer of the constituent parties, a substantial number of voters (more than one-third) score coalitions above or below the parties they are composed of, which indicates that at least for these voters coalitions are distinct political objects. Party-centered voters, that is, those with party identification, are less likely to score the coalition independently from the parties it is composed of. Yet, party identification for one of the coalition parties translates in a surplus preference for the coalition itself. Second, the different effect of political knowledge and education across coalitions and the limited significance of the two variables at the coalition level suggest that strong attitudes toward coalitions may not be solely the realm of the most sophisticated voters.

## 7. Conclusion and discussion

When the institutional setup fosters multiparty systems and coalition governments, voters have incentives to think beyond party voting and to consider post-electoral bargaining processes. Previous research suggests that voters have a rather good understanding of policy compromises and coalition formation at the post-electoral state (e.g. Hobolt and Karp 2010). The recent literature also demonstrates that the way voters evaluate potential coalition governments influences their voting behavior (e.g. Blais et al. 2006; Duch et al. 2010). Yet, we know surprisingly little about the *nature* of voters’ coalition preferences. Building on a recent strand of research which suggests a strong role of coalitions’ evaluations (Huber 2014; Meffert and Gschwend 2012; Meyer and Strobl 2016), we examined in detail the idea of preferences for coalitions as separate entities distinct from parties.

We investigated, first, what determines coalition preferences. Our findings provide support for the coalition-directed voting literature (Bargsted and Kedar 2009; Duch et al. 2010) indicating that voters think in terms of policy coalition outcomes above and beyond their ideological proximity to the coalition partners. Party and leader heuristics, however, strongly inform coalition attitudes. In addition, voters seem to care whether coalition governments will be able to govern effectively as assessed by looking at the heterogeneity in coalition partners’ positions. We also found that the evaluation of the junior coalition partner has a disproportionally large influence on coalition preferences. This fits with the results from Bowler et al. (2014), indicating a strong effect of the junior coalition partner on coalitions’ evaluations. Above all, we find that coalitions represent discrete political objects for many voters and they are not merely a function of existing party preferences. This is confirmed in a second empirical step where we find that for a substantial number of voters, coalition thermometer ratings are not just a composite of party thermometer ratings but they are either warmer or cooler.

These results have important consequences for our understanding of voting behavior and party politics. On the former, the finding that coalition evaluations are not just confined to sophisticated voters is good news for our understanding of a voters’ capability and the overall process of electoral democracy. The political compromise that coalitions may offer to voters, especially for those more interested in these compromises such as more moderate voters, can be seen as positive. When it comes to party politics, coalition thinking may have positive consequences by inducing party leaders to try to reconcile their differences, insofar as possible, before an election. By emphasizing certain coalition options before the elections, parties can ease voters’ burden in deciding upon which party to support.

Hopefully, data of the sort that were available to us from Austria will become available in other countries as well, allowing scholars to test whether these findings hold in other countries. In this regard, we find that the majority of voters is able to score their preferences for coalitions. Nevertheless, respondents find it more difficult to answer survey questions on unlikely coalitions arrangements (i.e. higher levels of non-response), as was the case for the, admittedly, very improbable SPÖ–FPÖ coalition. One might also explore the attitude formation processes of coalition preferences to provide a clearer theoretical foundation for the patterns we explored. Such a study, however, requires time-series or experimental data. Ours is just a first step in exploring the nature of coalition preferences as one of the important heuristics voters employ in their vote choice.

## Supplementary Material

OnlineAppendix_JEPOP-2015-0091_R_R3.doc

## Notes

1. Using distance to issue positions on immigration and/or the economy to calculate voter–party proximity instead of a general left–right ideological scale does not change substantive conclusions.

2. The fact that surveys do not usually ask voters to place expected governments on a left–right scale have until now meant that scholars had to rely on seat-weighted average of party positions to measure coalition positions (Duch et al. 2010; Kedar 2005, 2009). Recent findings, however, indicate that these calculations may not correspond to the reality (Bowler et al. 2014; Meyer and Strobl 2016). We thus rely on a measure of coalition positions as perceived by voters because ‘although voters may at times be mistaken about these locations, it is their personal beliefs … that will guide preference formation’ (Westholm 1997, 870; see also Kedar 2009; Blais et al. 2001). Note, however, that the same measure computed using the seat-weighted average of the two constituent parties instead of the position of the overall coalition leads to similar but weaker associations.

3. In all coalitions including the party SPÖ, this is the larger party and we consider preferences for the SPÖ party leader, Werner Faymann; for the coalition ÖVP–FPÖ, the ÖVP is the larger party and thus we consider the preferences for Michael Spindelegger.

4. Our analyses use the *xtmixed* command in Stata 14.

5. These transformations are discussed in detail in the Online Appendix.

6. We also test alternatives to this categorization. In particular, we looked at the ideological distance to the most and the least *liked* party and the distance to the most and least *distant* party within the coalition. The results are presented in the Online Appendix in Table S4 and Table S5, respectively.

7. In our models, we do not control for voters expectations over coalition outcomes. Existing studies in this regard refer to both a ‘bandwagon effect’ with expectations exerting a strong effect on preferences but also a ‘wishful thinking’ effect, with preferences strongly influencing expectations (e.g. Meffert et al. 2011; Huber 2014; Bartels 2002). Given that the data at hand do not allow us to assess causality, we avoid the addition of likelihood perceptions in our empirical models. It is important to stress, however, that the addition of a variable that measures likelihood perceptions in our models does not alter substantive conclusions as shown in Table S3 of the Online Appendix.

8. Table S2 in the Online Appendix presents correlation coefficients and indicates that collinearity levels are not troublesome.

9. Note that running models for each coalition arrangement separately would lead to similar substantive conclusions. The only interesting difference pertains to the values of *R*^2^ that are quite high and generally above 0.5 for the classical large–junior coalition arrangement, while we face a lower *R*^2^ for the grand coalition SPÖ–ÖVP.

10. The number of respondents in the regression tables is lower than the number shown in Table 3. In fact, the regression models can include only the respondents that have evaluated all four coalitions considered in the paper. Recreating Table 3 with only the respondents included in the regression models leads to almost identical patterns; see Table S8 in the Online Appendix. Also, our substantive conclusions are in line with those we would obtain imputing missing values as shown in Table S9.

11. Models use the *gsem* command in Stata 14.

12. Note that using party preferences instead of identification leads to very similar results but it somewhat diminishes the effect of all other variables.
