# Supplementary material for: On the nature of voters’ coalition preferences
Source: J Elect Public Opin Parties. 2016 Dec 26;27(3):254–73. doi: 10.1080/17457289.2016.1270286 (PMC5546046; doi:10.1080/17457289.2016.1270286)
Supplement: OnlineAppendix_JEPOP-2015-0091_R_R3.doc [file fbep_a_1270286_sm3042.doc]

**ONLINE APPENDIX**

**ON THE NATURE OF VOTERS’ COALITION PREFERENCES**

**1. Descriptive statistics of the variables used in the empirical models**

Table S1: Descriptive statistics of dependent and independent variables

|  | Mean | Std. Dev. | Min | Max |
| --- | --- | --- | --- | --- |
| Coalition preference | 3.602 | 3.081 | 0 | 10 |
| Parties preference similarity | 6.851 | 2.554 | 0 | 10 |
| Coalition includes top- ranked party | 0.429 | 0.495 | 0 | 1 |
| Coalition includes bottom-ranked party | 0.316 | 0.465 | 0 | 1 |
| Distance big party | 1.947 | 1.933 | 0 | 10 |
| Distance small party | 3.128 | 2.435 | 0 | 10 |
| Programmatic heterogeneity | 2.924 | 2.116 | 0 | 10 |
| Preference leader of big party | 4.763 | 2.699 | 0 | 10 |
| Leaders preference similarity | 7.013 | 2.510 | 0 | 10 |
| Distance to coalition | 2.308 | 2.061 | 0 | 10 |
| Incumbency | 0.262 | 0.440 | 0 | 1 |
| Education | 0.470 | 0.287 | 0 | 1 |
| Political knowledge | 1.850 | 0.883 | 0 | 7 |
| Extreme ideology | 1.471 | 1.443 | 0 | 5 |
| Education (*y-hat*) | -0.002 | 0.185 | -0.397 | 0.448 |
| Political knowledge (*y-hat*) | 0.001 | 0.053 | -0.176 | 0.119 |
| Extreme ideology (*y-hat*) | -0.001 | 0.366 | -1.494 | 0.795 |

Note: *N_stacked*=9689. (*y-hat*): because we deal with stacked data our models include linear transformations of the variables education, political knowledge and extremism. For details on how this is done see van der Eijk et al. (2006) and the discussion at page 7 of this supporting material.

**2. Matrix of correlation coefficients**

Table S2: Pearson correlations for main independent variables

|  | Distance to: | | | Coalition expectations | Programmatic heterogeneity | Preference similarity: | | Coalition includes party | |
| --- | --- | --- | --- | --- | --- | --- | --- | --- | --- |
|  | coalition | largest party | smaller party |  |  | Leaders | Parties | top-ranked | bottom-ranked |
| Distance to: |  |  |  |  |  |  |  |  |  |
| coalition | 1 |  |  |  |  |  |  |  |  |
| largest party | 0.507 | 1 |  |  |  |  |  |  |  |
| smaller party | 0.636 | 0.267 | 1 |  |  |  |  |  |  |
| Coalition expectations | -0.172 | -0.043 | -0.304 | 1 |  |  |  |  |  |
| Programmatic heterogeneity | 0.167 | 0.145 | 0.431 | -0.283 | 1 |  |  |  |  |
| Preference similarity: |  |  |  |  |  |  |  |  |  |
| Leaders | -0.027 | 0.049 | -0.092 | 0.154 | -0.281 | 1 |  |  |  |
| Parties | -0.039 | 0.069 | -0.119 | 0.158 | -0.332 | 0.657 | 1 |  |  |
| Coalition includes party: |  |  |  |  |  |  |  |  |  |
| top-ranked | -0.248 | -0.192 | -0.285 | 0.142 | 0.007 | -0.254 | -0.381 | 1 |  |
| bottom-ranked | 0.215 | 0.033 | 0.373 | -0.329 | 0.193 | -0.192 | -0.305 | -0.249 | 1 |
| Preference leader of large party | -0.199 | -0.382 | -0.069 | 0.065 | 0.000 | -0.281 | -0.222 | 0.279 | 0.015 |

Note: *N_stacked*=9689.

**3. Alternative models and specification of variables in the models**

Table S3 presents a model that also includes voters’ expectations about the likelihood of certain coalition arrangements taking place after the elections. *Coalition* *expectations* are measured using the question: “How likely do you think it is that the following parties will form a coalition after the election? Very likely, fairly likely, fairly unlikely or very unlikely?” As explained in footnote 7 of the paper, existing studies found evidence for both a ‘bandwagon effect’ with expectations exerting a strong effect on coalition preferences, our dependent variable, but also a ‘wishful thinking’ effect, with coalition preferences strongly influencing expectations (e.g. Meffert et al. 2011; Huber 2014; Bartels 2002). Given that the data at hand do not allow us to assess causality, and because by adding expectations we might incur in a problem of endogeneity, we avoid the addition of likelihood perceptions in our empirical models. Table S3, however, shows that the addition of a variable that measures likelihood perceptions in our models does not alter substantive conclusions.

We also present alternative specifications of two main variables in our models. In particular, instead of looking at the effect of the ideological distance to the largest and smallest coalition partner, we looked at the ideological distance to the most and the least *liked* party and the distance to the most and least *distant* party within the coalition. The results are presented in in Table S4 and Table S5 respectively. These additional results confirm our findings of a differentiated effect of the two coalition partners on the overall coalition preference. In particular, Table S4 shows that evaluations of the least liked coalition partner exert a stronger effect on coalition preferences compared to evaluations of the most liked coalition partner. Similarly Table S5 indicates that evaluations of the most distant coalition partner exert a stronger effect on coalition preferences compared to evaluations of the *least distant* coalition partner.

Table S3: The determinants of coalition preferences (with *coalition likelihood*): Hierarchical linear regression models

| Dependent variable: Coalition preference | | |
| --- | --- | --- |
|  | (M7) |  |
|  | Coefficient | Standard Error |
| Distance to coalition | -0.22*** | (0.02) |
| Programmatic heterogeneity | 0.07*** | (0.01) |
| Distance largest party | 0.03 | (0.02) |
| Distance smaller party | -0.22*** | (0.01) |
| Parties preference similarity | 0.11*** | (0.01) |
| Coalition includes top-ranked party | 1.72*** | (0.06) |
| Coalition includes bottom-ranked party | -0.83*** | (0.06) |
| Leaders preference similarity | 0.06*** | (0.01) |
| Preference leader of large party | 0.22*** | (0.01) |
| Education (*y-hat*) | 0.27* | (0.12) |
| Political knowledge (*y-hat*) | 0.35 | (0.42) |
| Extreme ideology (*y-hat*) | 0.01 | (0.07) |
| Coalition expectations | 0.80*** | (0.03) |
| Reference (SPÖ-Green coalition): |  |  |
| SPÖ-ÖVP | -0.34*** | (0.07) |
| ÖVP-FPÖ | -0.14* | (0.06) |
| SPÖ-FPÖ | -0.45*** | (0.08) |
| Intercept variance, *respondents* | 0.76 |  |
| *N_stacked (respondents*×*coalitions)* | 9399 |  |
| *N* (*respondents*) | 2653 |  |
| Log Likelihood | -20949.5 |  |
| AIC | 41937.00 |  |
| BIC | 42072.82 |  |

Notes: Standard errors in parentheses: * p<.05, ** p<.01, *** p<.001.

*y-hat* variables are predicted values.

Table S4: The determinants of coalition preferences (with *distance to the most/least liked party*): Hierarchical linear regression models

|  | Dependent variable: Coalition preference | | | | | |
| --- | --- | --- | --- | --- | --- | --- |
|  | (M1) | (M2) | (M3) | (M4) | (M5) | (M6) |
| Distance to coalition | -0.57*** |  |  | -0.36*** |  | -0.26*** |
|  | (0.01) |  |  | (0.01) |  | (0.02) |
| Programmatic heterogeneity | -0.05*** | 0.11*** |  | -0.03* | 0.06*** | 0.03 |
|  | (0.02) | (0.02) |  | (0.01) | (0.02) | (0.02) |
| Distance *most liked* party |  | -0.26*** |  |  | -0.14*** | -0.07*** |
|  |  | (0.02) |  |  | (0.02) | (0.02) |
| Distance *least liked* party |  | -0.42*** |  |  | -0.24*** | -0.13*** |
|  |  | (0.02) |  |  | (0.01) | (0.02) |
| Parties preference similarity |  |  | 0.14*** | 0.12*** | 0.13*** | 0.12*** |
|  |  |  | (0.02) | (0.01) | (0.02) | (0.02) |
| Coalition includes top-ranked party |  |  | 2.27*** | 2.02*** | 1.97*** | 1.94*** |
|  |  |  | (0.06) | (0.06) | (0.06) | (0.06) |
| Coalition includes bottom-ranked party |  |  | -1.33*** | -1.15*** | -1.13*** | -1.09*** |
|  |  |  | (0.07) | (0.06) | (0.06) | (0.06) |
| Leaders preference similarity |  |  | 0.06*** | 0.05*** | 0.05*** | 0.05*** |
|  |  |  | (0.01) | (0.01) | (0.01) | (0.01) |
| Preference leader of large party |  |  | 0.26*** | 0.21*** | 0.21*** | 0.20*** |
|  |  |  | (0.01) | (0.01) | (0.01) | (0.01) |
| Education (*y-hat*) | 0.74*** | 0.53*** | 0.41** | 0.31* | 0.21 | 0.24 |
|  | (0.15) | (0.15) | (0.13) | (0.13) | (0.13) | (0.13) |
| Political knowledge (*y-hat*) | 0.91 | 1.08* | 0.27 | 0.38 | 0.48 | 0.46 |
|  | (0.50) | (0.49) | (0.45) | (0.43) | (0.43) | (0.42) |
| Extreme ideology (*y-hat*) | 0.10 | 0.23** | 0.55*** | 0.08 | 0.19** | 0.04 |
|  | (0.08) | (0.08) | (0.07) | (0.07) | (0.07) | (0.07) |
| Reference (SPÖ-Green coalition): |  |  |  |  |  |  |
| SPÖ-ÖVP | 0.26*** | 0.23** | 0.44*** | 0.23*** | 0.25*** | 0.19** |
|  | (0.07) | (0.07) | (0.07) | (0.06) | (0.07) | (0.06) |
| ÖVP-FPÖ | -1.10*** | -1.01*** | -0.54*** | -0.46*** | -0.44*** | -0.44*** |
|  | (0.07) | (0.07) | (0.07) | (0.07) | (0.07) | (0.07) |
| SPÖ-FPÖ | -1.71*** | -1.64*** | -1.06*** | -1.11*** | -1.05*** | -1.12*** |
|  | (0.09) | (0.09) | (0.07) | (0.08) | (0.08) | (0.07) |
| Intercept | 5.67*** | 5.74*** | 0.73*** | 2.12*** | 2.07*** | 2.36*** |
|  | (0.07) | (0.07) | (0.15) | (0.16) | (0.16) | (0.16) |
| Intercept variance, *respondents* | 0.59 | 0.64 | 0.70 | 0.86 | 0.81 | 0.89 |
| *N_stacked (respondents*×*coalitions)* | 9689 | 9689 | 9689 | 9689 | 9689 | 9689 |
| *N* (*respondents*) | 2706 | 2706 | 2706 | 2706 | 2706 | 2706 |
| Log likelihood | -23302.7 | -23248.7 | -22397.9 | -22045.5 | -22108.7 | -21997.2 |
| AIC | 46627.3 | 46521.5 | 44823.8 | 44123.0 | 44251.5 | 44030.3 |
| BIC | 46706.3 | 46607.6 | 44924.3 | 44237.8 | 44373.5 | 44159.5 |

Note: as Table 2 in the paper but with distance to the most and least *liked* party rather than distance to the largest and smallest party. Standard errors in parentheses: * p<.05, ** p<.01, *** p<.001. *y-hat* variables are predicted values.

Table S5: The determinants of coalition preferences (with *distance to the most/least distant party*): Hierarchical linear regression models

|  | Dependent variable: Coalition preference | | | | | |
| --- | --- | --- | --- | --- | --- | --- |
|  | (M1) | (M2) | (M3) | (M4) | (M5) | (M6) |
| Distance to coalition | -0.57*** |  |  | -0.36*** |  | -0.25*** |
|  | (0.01) |  |  | (0.01) |  | (0.02) |
| Programmatic heterogeneity | -0.05*** | 0.17*** |  | -0.03* | 0.11*** | 0.04* |
|  | (0.02) | (0.02) |  | (0.01) | (0.02) | (0.02) |
| Distance *most distant* party |  | -0.52*** |  |  | -0.32*** | -0.16*** |
|  |  | (0.02) |  |  | (0.02) | (0.02) |
| Distance *least distant* party |  | -0.24*** |  |  | -0.11*** | -0.07** |
|  |  | (0.03) |  |  | (0.02) | (0.02) |
| Parties preference similarity |  |  | 0.14*** | 0.12*** | 0.13*** | 0.13*** |
|  |  |  | (0.02) | (0.01) | (0.01) | (0.01) |
| Coalition includes top-ranked party |  |  | 2.27*** | 2.02*** | 1.96*** | 1.94*** |
|  |  |  | (0.06) | (0.06) | (0.06) | (0.06) |
| Coalition includes bottom-ranked party |  |  | -1.33*** | -1.15*** | -1.08*** | -1.07*** |
|  |  |  | (0.07) | (0.06) | (0.06) | (0.06) |
| Leaders preference similarity |  |  | 0.06*** | 0.05*** | 0.05*** | 0.05*** |
|  |  |  | (0.01) | (0.01) | (0.01) | (0.01) |
| Preference leader of big party |  |  | 0.26*** | 0.21*** | 0.20*** | 0.20*** |
|  |  |  | (0.01) | (0.01) | (0.01) | (0.01) |
| Education (*y-hat*) | 0.74*** | 0.55*** | 0.41** | 0.31* | 0.24 | 0.25* |
|  | (0.15) | (0.14) | (0.13) | (0.13) | (0.13) | (0.13) |
| Political knowledge (*y-hat*) | 0.91 | 1.19* | 0.27 | 0.38 | 0.55 | 0.50 |
|  | (0.50) | (0.49) | (0.45) | (0.43) | (0.43) | (0.42) |
| Extreme ideology (*y-hat*) | 0.10 | 0.13 | 0.55*** | 0.08 | 0.13 | 0.02 |
|  | (0.08) | (0.08) | (0.07) | (0.07) | (0.07) | (0.07) |
| Reference (SPÖ-Green coalition): |  |  |  |  |  |  |
| SPÖ-ÖVP | 0.26*** | 0.24** | 0.44*** | 0.23*** | 0.26*** | 0.20** |
|  | (0.07) | (0.07) | (0.07) | (0.06) | (0.06) | (0.06) |
| ÖVP-FPÖ | -1.10*** | -0.99*** | -0.54*** | -0.46*** | -0.44*** | -0.44*** |
|  | (0.07) | (0.07) | (0.07) | (0.07) | (0.07) | (0.06) |
| SPÖ-FPÖ | -1.71*** | -1.66*** | -1.06*** | -1.11*** | -1.09*** | -1.13*** |
|  | (0.09) | (0.08) | (0.07) | (0.08) | (0.08) | (0.07) |
| Intercept | 5.67*** | 5.93*** | 0.73*** | 2.12*** | 2.22*** | 2.41*** |
|  | (0.07) | (0.07) | (0.15) | (0.16) | (0.16) | (0.16) |
| Intercept variance, *respondents* | 0.59 | 0.77 | 0.70 | 0.86 | 0.84 | 0.91 |
| *N_stacked (respondents*×*coalitions)* | 9689 | 9689 | 9689 | 9689 | 9689 | 9689 |
| *N* (*respondents*) | 2706 | 2706 | 2706 | 2706 | 2706 | 2706 |
| Log likelihood | -23302.7 | -23180.6 | -22397.9 | -22045.5 | -22089.2 | -21994.7 |
| AIC | 46627.3 | 46385.3 | 44823.8 | 44122.9 | 44212.3 | 44025.4 |
| BIC | 46706.3 | 46471.4 | 44924.3 | 44237.8 | 44334.4 | 44154.6 |

Note: as Table 2 in the paper but with distance to the most and least *distant* party rather than distance to the largest and smallest party. Standard errors in parentheses: * p<.05, ** p<.01, *** p<.001. *y-hat* variables are predicted values.

**4. Discussion of the *y-hat* variables**

As one can assume that parties’ and coalitions’ evaluations have effects on all potential coalitions and not only on particular ones, modeling all potential coalitions in one model offers a general picture of the effect of these evaluations. Thus, for our analysis, the data set is expanded by the number of coalitions available in our survey. In this ‘stacked’ dataset, each respondent contributes four observations, one for each of the coalitions available in our survey; for details on this process, see for example Pardos-Prado and Dinas (2010).

This reshaping changes the unit of analysis from the number of individual respondents (*N*) to *N*×*C* respondent–coalitions (van der Eijk et al. 2006). Because of this, an independent variable in order to be included in the analysis also needs to be defined in terms of bivariate relationships between the chooser (i.e., respondent) and the object of evaluation (i.e., coalition). Some independent variables such as left-right voter/coalition proximity are already defined as respondent×coalition specific relationships. Other variables instead, like socio-demographic variables, need to be re-conceptualized as proximity measure in order to capture the empirical relationship between voters and coalitions. In order to include the variables education, political knowledge and extreme ideology in our models, we apply the so-called ‘*y-hat*’ approach ([van der Eijk and Franklin](#bookmark374) [1996](#bookmark374)). The specific procedure for doing so is based on multivariate regressions (run separately for each coalition) on the specific predictors: predicted values (*y-hats*) are then centered on their means and saved as scores for use in the later analysis as coalition-respondent-specific predictors (De Sio and Franklin 2007).[[1]](#footnote-2)

For *y-hats* in particular, the b coefficient for a specific predictor (e.g., political knowledge) does not express the effect of the particular indicator, but rather the importance of, e.g., political knowledge in general (in this, *y-hats* are rather like factor scores, but tuned to provide the best available linear prediction of the dependent variable). So b coefficients for *y-hat* measures are always positive (in the absence of collinearity with other predictors they would all equal 1.0), and (with regard to multicollinearity) behave rather like beta coefficients (effects of standardized variables). The *y-hats* contain exactly the same explanatory information as the original independent variable(s) as they are nothing else than a linear transformation of the original variable(s).

Usually*, y-hat* affinities are used only as control variables, in contrast to the coalition–respondent issue affinities already described, whose values are the theoretically called-for product of respondent and coalition scores. Given however the substantive importance of the variables education, political knowledge and extreme ideology for our analysis, to be able to interpret both the significance and the direction of the coefficient we report below the effects of the same predictors in different stacks, i.e., coalitions, obtained specifying the option “effects” in the “*genyhats*” command of the *ptvtools* package. The “*genyhats*” command is followed by the “*iimpute*” command whose primary purpose is that of imputing missing values for the battery of *y-hat* items. This command is available within the *ptvtools* package as well.

Table S6 presents the linear regression coefficients of education, political knowledge and extremism by coalition. These are the coefficients then used to calculate the *y-hat* variables used in Table 2 in the paper. Table S7 presents the logistic regression coefficients of education, political knowledge and extremism by coalition. These are the coefficients then used to calculate the *y-hat* variables used in Table 4 in the paper.

Table S6: Linear regression coefficients of education, political knowledge and extremism by coalition

|  | Dependent variable: Coalition preference | | | |
| --- | --- | --- | --- | --- |
|  | SPÖ-Green | SPÖ-ÖVP | ÖVP-FPÖ | SPÖ-FPÖ |
| Education | 0.76*** | -0.38* | -0.74*** | -0.63*** |
|  | (0.20) | (0.18) | (0.18) | (0.17) |
| Constant | 3.70*** | 4.92*** | 3.11*** | 2.53*** |
|  | (0.11) | (0.10) | (0.10) | (0.09) |
| *N* (*respondents*) | 2997 | 3035 | 2993 | 2927 |
| Adj. R-squared | 0.004 | 0.001 | 0.005 | 0.005 |
| Political knowledge | 0.10 | 0.03 | -0.05 | -0.03 |
|  | (0.06) | (0.06) | (0.06) | (0.05) |
| Constant | 3.87*** | 4.68*** | 2.87*** | 2.29*** |
|  | (0.13) | (0.12) | (0.12) | (0.11) |
| *N* (*respondents*) | 3010 | 3049 | 3006 | 2941 |
| Adj. R- squared | 0.000 | 0.000 | 0.000 | 0.000 |
| Extreme ideology | -0.22*** | -0.42*** | -0.12** | -0.11** |
|  | (0.04) | (0.04) | (0.04) | (0.03) |
| Constant | 3.77*** | 5.37*** | 2.92*** | 2.39*** |
|  | (0.08) | (0.07) | (0.07) | (0.07) |
| *N* (*respondents*) | 2866 | 2898 | 2865 | 2801 |
| Adj. R- squared | 0.010 | 0.045 | 0.003 | 0.004 |

Notes: Coefficients obtained using the "*effects*" option of the "*genyats*" command in the *ptvtolls* package. These coefficients are those used to calculate the *y-hat* variables used in Table 2.

Table S7: Logit coefficients of education, political knowledge and extremism by coalition

|  | Coalition score: (Reference category: Coalition as (weighted) average of the constituent parties) | | | | | | | |
| --- | --- | --- | --- | --- | --- | --- | --- | --- |
|  | above top party | | | | below bottom party | | | |
|  | SPÖ-Green | SPÖ-ÖVP | ÖVP-FPÖ | SPÖ-FPÖ | SPÖ-Green | SPÖ-ÖVP | ÖVP-FPÖ | SPÖ-FPÖ |
| Education | 0.08** | 0.01 | 0.06** | -0.01 | 0.06* | 0.04 | -0.05 | -0.01 |
|  | (0.03) | (0.03) | (0.02) | (0.02) | (0.03) | (0.03) | (0.03) | (0.03) |
| Constant | 0.02*** | 0.23*** | 0.15*** | 0.09*** | 0.30*** | 0.24*** | 0.30*** | 0.35*** |
|  | (0.11) | (0.02) | (0.01) | (0.01) | (0.02) | (0.02) | (0.02) | (0.02) |
| *N* (*respondents*) | 2132 | 2379 | 2248 | 1989 | 2596 | 2484 | 2726 | 2744 |
| Adj. R- squared | 0.003 | 0.000 | 0.003 | 0.000 | 0.001 | 0.000 | 0.001 | 0.000 |
| Political knowledge | -0.02* | 0.01 | -0.02* | -0.003 | -0.02* | -0.02 | -0.01 | -0.02 |
|  | (0.01) | (0.01) | (0.01) | (0.01) | (0.01) | (0.01) | (0.01) | (0.01) |
| Constant | 0.23*** | 0.22*** | 0.15*** | 0.10*** | 0.38*** | 0.29*** | 0.30*** | 0.38*** |
|  | (0.02) | (0.02) | (0.02) | (0.01) | (0.02) | (0.02) | (0.02) | (0.02) |
| *N* (*respondents*) | 2140 | 2388 | 2257 | 1999 | 2609 | 2495 | 2736 | 2757 |
| Adj. R- squared | 0.002 | 0.000 | 0.001 | 0.000 | 0.002 | 0.001 | 0.000 | 0.001 |
| Extreme ideology | 0.02*** | -0.03*** | -0.0003 | -0.01* | -0.03*** | -0.01 | -0.02*** | -0.05*** |
|  | (0.01) | (0.01) | (0.01) | (0.004) | (0.01) | (0.01) | (0.01) | (0.01) |
| Constant | 0.15*** | 0.28*** | 0.12*** | 0.11*** | 0.37*** | 0.28*** | 0.30*** | 0.41*** |
|  | (0.01) | (0.01) | (0.01) | (0.01) | (0.01) | (0.01) | (0.01) | (0.01) |
| *N* (*respondents*) | 2041 | 2276 | 2156 | 1910 | 2489 | 2375 | 2616 | 2628 |
| Adj. R- squared | 0.006 | 0.013 | 0.000 | 0.002 | 0.008 | 0.001 | 0.005 | 0.021 |

Notes: Coefficients obtained using the "*effects*" option of the "*genyats*" command in the *ptvtolls* package. These coefficients are those used to calculate the *y-hat* variables used in Table 4.

**5. Discussion of missing values**

The number of respondents in the regression tables in the paper is lower than the number shown in Table 3. In fact, our regression models can only include those respondents who answered all (four) coalition preference questions considered in the paper. We also lose additional respondents due to missing values in our main independent variables. Recreating Table 3 with only the respondents included in the estimations leads to almost identical patterns as shown in Table S8 below. Second, our substantive conclusions in Table 2 are in line with those we would obtain imputing missing values as shown in Table S9. Imputed values are obtained using the “mi estimate” command in Stata 14 using the data augmentation (DA) algorithm, which belongs to the family of MCMC procedures. The algorithm fills missing data by drawing from a conditional distribution, in this case, a multivariate normal, given the observed data. Simulation studies have shown that assuming a MVN distribution leads to reliable estimates in most cases, even when the normality assumption is violated when sample size is sufficiently large (Demirtas et al. 2008; Lee and Carlin 2010). However, since missing data analyses are always problematic as there is no inherently correct methodological procedure, we prefer not to impute missing data in our paper.

Table S8: Patterns of coalition preferences, including only respondents included in the regression models (cell entries are column %)

|  | COALITION | | | |
| --- | --- | --- | --- | --- |
|  | SPÖ-Greens | SPÖ-ÖVP | ÖVP-FPÖ | SPÖ-FPÖ |
| Coalition score somewhat in the middle or as high (low) as top (bottom) party | 59.3 | 61.4 | 68.1 | 63.9 |
| Coalition score above top party | 13.0 | 17.8 | 8.7 | 6.1 |
| Coalition score below bottom party | 27.7 | 20.8 | 23.2 | 30.0 |
| *N_stacked (respondents*×*coalitions)* | 9689 | 9689 | 9689 | 9689 |

Table S9: The determinants of coalition preferences: Hierarchical linear regression models (after imputation)

|  | Dependent variable: Coalition preference | | | | | |
| --- | --- | --- | --- | --- | --- | --- |
|  | (M1) | (M2) | (M3) | (M4) | (M5) | (M6) |
| Distance to coalition | -0.54*** |  |  | -0.35*** |  | -0.23*** |
|  | (0.01) |  |  | (0.01) |  | (0.02) |
| Programmatic heterogeneity | -0.06*** | 0.11*** |  | -0.03* | 0.07*** | 0.04** |
|  | (0.01) | (0.01) |  | (0.01) | (0.01) | (0.04) |
| Distance largest party |  | -0.20*** |  |  | -0.04** | 0.05** |
|  |  | (0.01) |  |  | (0.01) | (0.02) |
| Distance smaller party |  | -0.49*** |  |  | -0.33*** | -0.23*** |
|  |  | (0.01) |  |  | (0.01) | (0.01) |
| Parties preference similarity |  |  | 0.13*** | 0.11*** | 0.12*** | 0.11*** |
|  |  |  | (0.01) | (0.01) | (0.01) | (0.01) |
| Coalition includes top-ranked party |  |  | 2.16*** | 1.92*** | 1.79*** | 1.77*** |
|  |  |  | (0.06) | (0.06) | (0.06) | (0.06) |
| Coalition includes bottom-ranked party |  |  | -1.25*** | -1.08*** | -0.89*** | -0.89*** |
|  |  |  | (0.06) | (0.06) | (0.06) | (0.06) |
| Leaders preference similarity |  |  | 0.07*** | 0.06*** | 0.07*** | 0.07*** |
|  |  |  | (0.01) | (0.01) | (0.01) | (0.01) |
| Preference leader of large party |  |  | 0.26*** | 0.21*** | 0.25*** | 0.24*** |
|  |  |  | (0.01) | (0.01) | (0.01) | (0.01) |
| Education (*y-hat*) | 0.68*** | 0.45*** | 0.39** | 0.30** | 0.20 | 0.21 |
|  | (0.13) | (0.13) | (0.12) | (0.11) | (0.11) | (0.11) |
| Political knowledge (*y-hat*) | 0.78 | 0.88* | 0.28 | 0.34 | 0.46 | 0.42 |
|  | (0.44) | (0.43) | (0.40) | (0.39) | (0.39) | (0.38) |
| Extreme ideology (*y-hat*) | 0.15* | 0.20** | 0.51*** | 0.11 | 0.16** | 0.05 |
|  | (0.07) | (0.07) | (0.06) | (0.06) | (0.06) | (0.06) |
| Reference (SPÖ-Green coalition): |  |  |  |  |  |  |
| SPÖ-ÖVP | 0.38*** | 0.26*** | 0.53*** | 0.35*** | 0.30*** | 0.25*** |
|  | (0.07) | (0.07) | (0.06) | (0.06) | (0.06) | (0.06) |
| ÖVP-FPÖ | -0.99*** | -0.85*** | -0.50*** | -0.43*** | -0.37*** | -0.37*** |
|  | (0.07) | (0.07) | (0.06) | (0.06) | (0.06) | (0.06) |
| SPÖ-FPÖ | -1.66*** | -1.59*** | -1.10*** | -1.11*** | -1.08*** | -1.12*** |
|  | (0.08) | (0.07) | (0.06) | (0.07) | (0.07) | (0.07) |
| Intercept | 5.43*** | 5.61*** | 0.66*** | 2.03*** | 1.73*** | 1.98*** |
|  | (0.06) | (0.06) | (0.14) | (0.15) | (0.15) | (0.15) |
| *Random effects* |  |  |  |  |  |  |
| Intercept variance, *respondents* | 0.80 | 0.90 | 0.88 | 0.94 | 0.91 | 0.93 |
| *N_stacked (respondents*×*coalitions)* | 12006 | 12006 | 12006 | 12006 | 12006 | 12006 |
| *N* (*respondents*) | 3088 | 3088 | 3088 | 3088 | 3088 | 3088 |

Notes: Standard errors in parentheses: * p<.05, ** p<.01, *** p<.001. Imputation obtained using the *mi estimate* command in Stata 14 using the data augmentation (DA) algorithm.

**6. SURVEY QUESTIONS** (*full questionnaire available at:* [*http://www.autnes.at/?q=node/42*](http://www.autnes.at/?q=node/42)*)*

**English version**

Q58 Now, I'd like to ask you a few questions about the next federal government. Using a scale from 0 to 10, please indicate to what extent you would prefer a coalition between the following parties. 0 means ”I do not prefer this coalition at all” and 10 means ”I very much prefer this coalition”.

*[RANDOMISE ITEMS]*

Item 1 red-green, meaning SPÖ and the Greens

Item 2 red-black, meaning SPÖ and ÖVP

Item 3 black-blue, meaning ÖVP and FPÖ

Item 4 red-blue, meaning SPÖ and FPÖ

I do not prefer it at all 0

to

I very much prefer it 10

don’t know 88

refused 99

Q59 How likely do you think it is that the following parties will form a coalition after the election? Very likely, fairly likely, fairly unlikely or very unlikely?

*[items in the same order as in question Q58]*

Item 1 red and green, meaning SPÖ and the Greens

Item 2 red and black, meaning SPÖ and ÖVP

Item 3 black and blue, meaning ÖVP and FPÖ

Item 4 red-blue, meaning SPÖ and FPÖ

very likely 1

fairly likely 2

fairly unlikely 3

very unlikely 4

don’t know 88

refused 99

Q60 Where would you place the following potential coalitions on a scale from 0 to 10, where 0 means “left” and 10 means “right”? You can use the values in between to give a more precise answer.

*[items in the same order as in question Q58]*

Item 1 red and green, meaning SPÖ and the Greens

Item 2 red and black, meaning SPÖ and ÖVP

Item 3 black and blue, meaning ÖVP and FPÖ

Item 4 red-blue, meaning SPÖ and FPÖ

left 0

to

right 10

don’t know 88

refused 99

**German version**

Q58 Jetzt habe ich ein paar Fragen zur nächsten Bundesregierung. Wie sehr wünschen Sie sich eine Koalition zwischen den folgenden Parteien? Bitte verwenden Sie hier wieder eine Skala von 0 bis 10 - wobei 0 bedeutet, diese Koalition wünsche ich mir überhaupt nicht, und 10 bedeutet, diese Koalition wünsche ich mir sehr.

*[ITEMS RANDOMISIEREN]*

Item 1 rot-grün, also SPÖ und Grüne

Item 2 rot-schwarz, also SPÖ und ÖVP

Item 3 schwarz-blau, also ÖVP und FPÖ

Item 4 rot-blau, also SPÖ und FPÖ

wünsche ich mir überhaupt nicht 0

bis

wünsche ich mir sehr 10

weiß nicht 88

verweigert 99

Q59 Und jetzt zur Wahrscheinlichkeit von Koalitionen. Für wie wahrscheinlich halten Sie es, dass die folgenden Parteien nach der Wahl eine Koalition bilden? Sehr wahrscheinlich, eher wahrscheinlich, eher unwahrscheinlich oder sehr unwahrscheinlich?

*[ITEMS IN DER GLEICHEN REIHENFOLGE WIE BEI FRAGE Q58]*

Item 1 rot und grün, also SPÖ und Grüne

Item 2 rot und schwarz, also SPÖ und ÖVP

Item 3 schwarz und blau, also ÖVP und FPÖ

Item 4 rot-blau, also SPÖ und FPÖ

sehr wahrscheinlich 1

eher wahrscheinlich 2

eher unwahrscheinlich 3

sehr unwahrscheinlich 4

weiß nicht 88

verweigert 99

Q60 Wo würden Sie folgende mögliche Koalition auf einer Skala von 0 bis 10 einordnen, wobei 0 "links" bedeutet und 10 "rechts" bedeutet? Mit den Werten dazwischen können Sie Ihre Einschätzung abstufen.

*[ITEMS IN DER GLEICHEN REIHENFOLGE WIE BEI FRAGE Q58]*

Item 1 rot-grün, also SPÖ und Grüne

Item 2 rot-schwarz, also SPÖ und ÖVP

Item 3 schwarz-blau, also ÖVP und FPÖ

Item 4 rot-blau, also SPÖ und FPÖ

links 0

bis

rechts 10

weiß nicht 88

verweigert 99

**REFERENCES**

Bartels, L. M. 2002. “The Impact of Candidate Traits in American Presidential Elections.” In *Leaders’ Personalities and the Outcomes of Democratic Elections*, Anthony King (ed.), 44-69. Oxford: Oxford University Press.

De Sio, L., and M. N. Franklin. 2011. “PTVTOOLS: A Stata package for PTV analysis (version 0.9)”, Statistical Software Components, Boston College

De Sio, L., and M. N. Franklin. 2012. “Strategic incentives, issue proximity and party support in Europe”. *West European Politics* 35(6): 1363-1385.

Demirtas, H., S. A. Freels, and R. M. Yucel. 2008. “Plausibility of multivariate normality assumption when multiply imputing non-Gaussian continuous outcomes: a simulation assessment”. *Journal of Statistical Computation and Simulation* 78(1): 69-84.

Huber, S. 2014. Coalitions and Voting Behavior in a Differentiating Multiparty System. In *Voters on the Move or on the Run?*, B. Wessels, H. Rattinger, S., Rossteutscher, and R., Schmitt-Beck (Eds.), 65–87. Oxford: Oxford University Press.

Lee, K. J., and J. B. Carlin. 2010. “Multiple imputation for missing data: fully conditional specification versus multivariate normal imputation”. *American Journal of Epidemiology* 171(5): 624-632.

Meffert, M.F., S. Huber, T. Gschwend, and F. U. Pappi. 2011. “More than wishful thinking: Causes and consequences of voters’ electoral expectations about parties and coalitions. ” *Electoral Studies* 30 (4): 804–815.

Pardos-Prado, S., and E. Dinas. 2010. “Systemic polarisation and spatial voting.” *European Journal of Political Research* 49 (6): 759-786.

van der Eijk, C. and M.N. Franklin. 1996. *Choosing Europe? The European Electorate and National Politics in the Face of the Union*. Ann Arbor, MI: The University of Michigan Press.

Van der Eijk, C., W. van der Brug, M. Kroh, and M. Franklin. 2006. “Rethinking the Dependent Variable in Voting Behavior: On the Measurement and Analysis of Electoral Utilities.” *Electoral Studies* 25 (3): 424–47.

1. In Stata we have used the *ptvtools* package (De Sio and Franklin 2011). [↑](#footnote-ref-2)
